# Poisoning by Strychnine

**Published:** 1879-01

**Authors:** V. R. Bridges


### NOTES FROM PRIVATE PRACTICE.

### ARTICLE VI.

*Poisoning by Strychnine.*

On the morning of May 12, 1878, I was called to see a little girl, aged 9 years, who—as the messenger stated—was having spasms. Supposing it to be a case of convulsions from intestinal irritation, or other ordinary cause, I made no extra preparations, but at once obeyed the summons.

On my arrival I was quite surprised to find myself confronted with a very serious case—such as I had never seen before. By following a proper line of inquiry I was not long in arriving at a diagnosis, which, notwithstanding the protestations of the family to the contrary, was fully confirmed in a few hours by finding an old strychnia bottle with a few grains of the salt yet in it, and which the child afterward admitted she had handled. Any estimate of the amount she took, of course would be conjectural, and I will not give a detailed history of the symptoms. Suffice it to say that for four hours before I saw her, and for nearly ten hours afterward, she was in a continuous and persistent spasm, from head to foot, with an alarming and distressing opisthotonos, rendered all the more painful from the fact that she was entirely conscious of her suffering, while any effort to relieve her by the slightest touch, or causing her to swallow, only aggravated her condition, by apparently exciting every muscle to increase its tension.

But the treatment adopted is the main object I have in view in reporting the case. I first prescribed .03 of morphia and applied hot water to the spine, but fearing the morphia alone would not be sufficient, I sent back to my office (5 miles) for other remedies, in the meantime continuing the hot water to the spine and, repeating the morphia every half hour. One and a half hours elapsed and, nothing gained, I then gave:

| | | |
|---|---|---|
| Flu. ex. belladonna | 2 | |
| Flu. ex. c. indic | 2 | |
| Morph. sulph | | 03 |
| Elix. simpl | 32 | |

M. Teaspoonful every hour alternated with the following:

| | |
|---|---|
| Potassic bromide | 6 |
| Hydr. chloral, | 3 |
| Elix. simpl | 32 |

M. Teaspoonful every hour.

This, though somewhat heroic, as I thought, for a small child, failed to relieve the patient as promptly as I desired, and I had almost despaired of her life, when an expression in one of Brown-Séquard's lectures occurred to me, in which he affirms that "Spasmodic action is due to unrest or a morbid activity of the cells of gray matter in the spinal cord and base of the brain, which results in the destruction of tissue and the exhaustion of nerve force," and he suggests as a remedy, friction, bandaging, or some other force or irritant applied to the extremities.

Acting upon this suggestion—in the midst of a severe convulsion—I grasped her thighs in my hands and used all the force I could in constricting them, when to my surprise and gratification she came promptly out of the spasm, and to some extent was relieved of the muscular stiffness and opisthotonos. To test this more fully, after a few minutes, I relinquished my grasp, and quick as thought she was in another spasm which was just as promptly relieved as before. Convinced of the value of this remedy, I detailed two men to keep up this pressure almost continuously for about four hours, thus preventing, as I now believe, "the destruction of nerve tissue" and brain force, and the consequent exhaustion of vital energy, until I could get my patient under the influence of powerful anodynes and nerve sedatives, and thus gain time, for I was now persuaded that time, and time only, under such influences, could save her.

To this end I persevered in the treatment—internal and external—alternating doses every 30 minutes, and giving the patient my own personal attention during the entire night.

At 5 a. m. on the 13th, I left my patient, with treatment all suspended, in a profound sleep, from which she awoke in about 6 hours, every way improved, and, save a little twitching of the muscles, at times, and some general muscular soreness, was free from the effect of the poison.

The point of special interest to me in the treatment of the case, was the promptness with which the patient was relieved by the suggestion as indicated in Brown-Sequard's lecture. There is no question in my mind but that it contributed largely to the successful issue of the case.

V. R. Bridges, m. d.

